# Contrast-Enhanced Ultrasound Reveals Exercise-Induced Perfusion Deficits in Claudicants

**DOI:** 10.21767/2573-4482.100041

**Published:** 2017-03-06

**Authors:** Rishi Kundi, Steven J Prior, Odessa Addison, Michael Lu, Alice S Ryan, Brajesh K Lal

**Affiliations:** 1 Department of Surgery, Division of Vascular Surgery, Baltimore VA Medical Center, University of Maryland School of Medicine, Baltimore, USA; 2 Department of Veterans Affairs and Baltimore Veterans Affairs Medical Center Geriatric Research, Education and Clinical Center (GRECC), USA; 3 Department of Medicine, Division of Gerontology and Geriatric Medicine, University of Maryland School of Medicine, Baltimore, USA

**Keywords:** Peripheral arterial disease, Contrast ultrasound, Claudication, Chronic peripheral ischemia, Perfusion, Vascular surgery

## Abstract

**Background:**

Contrast-Enhanced Ultrasonography (CEUS) is an imaging modality allowing perfusion quantification in targeted regions of interest of the lower extremity that has not been possible with color-flow imaging or with measurement of ankle brachial indices. We developed a protocol to quantify lower extremity muscle perfusion impairment in PAD patients in response to exercise.

**Methods and findings:**

Thirteen patients with Rutherford Class I-III Peripheral Arterial Disease (PAD) and no prior revascularization procedures were recruited from the Baltimore Veterans Affairs Medical Center and compared with eight control patients without PAD. CEUS interrogation of the index limb gastrocnemius muscle was performed using an intravenous bolus of lipid-stabilized microsphere contrast before and after a standardized treadmill protocol. Peak perfusion (PEAK) and time to peak perfusion (TTP) were measured before and after exercise. Between and within group differences were assessed. Control subjects demonstrated a more rapid TTP (p<0.01) and an increase in peak perfusion (PEAK, p=0.02) after exercise, when compared to their baseline measures. Patients with PAD demonstrated TTP and PEAK measures equivalent to controls at baseline (p=0.39, p=0.71, respectively). However, they exhibited no significant exercise-induced changes in perfusion (TTP p=0.49 and PEAK 0.67, respectively compared to baseline). After exercise, normal subjects had significantly shorter TTP (p=0.04) and greater PEAK (p=0.02) than PAD patients.

**Conclusion:**

Consistent with their lack of ischemic symptoms at rest, class I to III claudicant PAD patients showed similar perfusion measures (TTP and PEAK) at rest. PAD patients, however, were unable to increase perfusion in response to exercise, whereas controls increased perfusion significantly. This corresponds with claudication and limited walking capacity observed in PAD. CEUS with bolus injection offers a convenient, objective, quantitative and visual physiologic assessment of perfusion limitation in specific muscle groups of PAD patients. This has the potential for substantial clinical and research utility.

## Introduction

Contrast-Enhanced Ultrasound (CEUS) facilitates increasing the dynamic resolution of ultrasonography using a suspension of microscopic bubbles stabilized within a shell [1]. The composition of the shell varies but the most commonly used contrast uses lipid. The bubbles vary between one to five micrometers in diameter; smaller than erythrocytes the size of which is between six and eight micrometers. The bubbles are able to traverse the microcirculation but cannot cross the endothelium [2]. CEUS is thus able to display dynamic perfusion imagery down to the microvascular scale and in the context of surrounding tissue.

CEUS is able to reveal cardiac shunts, improve ventricular luminal surface visualization, and even confirm myocardial viability when used in echocardiography [3,4]. It can also determine perfusion of end-organs such as the kidney, and allows ultrasound-based distinction of hepatic tumor from normal liver parenchyma based on flow patterns [5–7]. CEUS has also been used to quantify angiogenesis [8,9] has been used by other groups to assess skeletal muscle perfusion. It is able to detect the inflammatory hyperemia of myositis and to demonstrate perfusion changes resulting from resistance training [10,11] of particular interest is the ability of CEUS to assess lower extremity perfusion in Peripheral Arterial Disease (PAD). Past studies have used CEUS to demonstrate resting perfusion deficits in PAD patients as well as to reveal perfusion changes following revascularization [12–15].

PAD affects more than two hundred million people globally and its incidence has increased more than 75% in the last 25 years [16]. As the elderly proportion of the population increases this will only increase. PAD afflicts patients in a spectrum represented by grading systems such as the Rutherford chronic limb ischemia classification system [17]. The Rutherford system spans Category 0 (asymptomatic) to Category 6 (limb gangrene). Categories 1 through 3 represent intermittent claudication or effort-induced ischemic pain of the lower extremity.

Currently, non-invasive diagnosis and assessment of PAD relies upon the measurement of the pressure at which the distal arteries of the lower extremity occlude in relation to the systemic blood pressure. The Ankle-to-Brachial Index (ABI) is a rapid method of detecting lower than normal perfusion pressures [18]. Because the ischemia of claudicants is effort-induced, the resting ABI in these patients is often normal with the addition of provocative exercise, however, the presence of PAD is unmasked, and the exercise ABI in claudicants can be lower than expected [19]. These studies, while convenient do not actually measure perfusion of specific lower extremity musculature but only the pressure within the named arteries of the leg. CEUS offers an opportunity to image the end-organ perfusion of the lower extremities.

Investigations of this potential application of CEUS to PAD have been encouraging. At rest, CEUS has shown that blood flow to the gastrocnemius in PAD patients is significantly different than in normal controls, and different in diabetic patients without clinically significant PAD [12,20]. As with ABI, however, the nature of claudication makes resting perfusion assessment unreliable. Transient occlusion has been used to cause a reactive vasodilation and hyperemia similar to that provoked by effort. CEUS of post-occlusive reactive hyperemia has been used in healthy volunteers as well as PAD patients and asymptomatic diabetics [15,20,21]. The validity of post-occlusive hyperemia as a surrogate for exercise, however, is questionable; perfusion after transient occlusion correlates with perfusion after submaximal exercise, not maximal and the clinical safety of tourniquet use on a patient with PAD is uncertain [22].

Therefore, we investigated the utility of CEUS for assessing perfusion deficits in PAD patients in response to a standardized bout of walking exercise. In this pilot study, patients with Class I-III chronic peripheral arterial disease and normal control subjects underwent bolus injections of ultrasound contrast and gastrocnemius perfusion assessment before and after provocative exercise using a graded treadmill protocol.

## Materials and Methods

### Study population

Patients with an existing diagnosis of PAD and symptoms of intermittent claudication were recruited from the outpatient vascular surgery clinic at the Baltimore Veterans Affairs Medical Center. All participants were without rest pain, open wounds or gangrene. Patients were independently ambulatory and had no contraindication to exercise testing. Pertinent medical histories and imaging studies were gathered by self-report, interview and medical record review. For purposes of comparison, eight subjects with palpable pedal pulses, no diagnosis of PAD and no other lower extremity symptoms volunteered as controls. Informed consent was obtained from all patients. All study procedures were approved by the institutional review board of the University of Maryland School of Medicine. The study protocol conformed to the 1975 Declaration of Helsinki.

### Procedures protocol

Subjects were first given a baseline CEUS examination. Following this, they were asked to lie supine for twenty to 25 min in order to ensure that a minimum of 30 min would separate contrast bolus injections. Graded treadmill exercise was then performed. The time of onset of claudication was noted. When the subject reported maximal, limiting pain, the treadmill was stopped and the subject was positioned supine for repeat CEUS examination. Control patients stopped treadmill exercise after 10 min. Post-exercise CEUS examination was then performed. All images were acquired digitally in both still and cine formats.

## Graded Treadmill Exercise Test

Prior to imaging, maximal walking capacity was assessed by graded treadmill testing. Subjects began treadmill walking at 2 mph or their normal gait speed, if it was less than 2 mph [23]. The initial treadmill grade was set at zero but was increased by 2% every 2 min. The maximum grade, speed, claudication onset time, maximal walking distance, distances of claudication onset and of onset of limiting pain were recorded. Treadmill speed and grade at test termination (the point of maximal claudication pain) were recorded for determination of the workload used during exercise testing with CEUS. This testing was performed at least one day prior to CEUS exercise testing.

### Ultrasound contrast

Perflutren lipid-stabilized microsphere ultrasound contrast (DEFINITY^®^, Lantheus, and Billerica, MA) was used for the study. One 1.5 mL vial was used for each examination. Vials were stored in a refrigerated unit prior to use and were agitated for one to 2 min before being drawn into a 1 mL Tuberculin syringe in a weight-based dose (10 μL/kg) with a maximal bolus dose of 0.75 mL. Contrast was administered through an upper extremity, peripheral intravenous line followed by a 10 mL normal saline flush. Patients received one bolus of contrast prior to exercise for basal perfusion assessment and another bolus immediately following treadmill walking for exercise perfusion assessment, with a minimum of 30 min between boluses of contrast. No subject was administered more than 1.5 mL of contrast.

### Ultrasound examination

Subjects were positioned supine on a stretcher. The index limb (or the leg that experienced more severe claudication in case PAD was bilaterally symptomatic) was flexed and externally rotated. The level of the calf with the greatest circumference was determined and marked. The circumference was recorded at that location, as was its distance from the medial malleolus and the tibial tubercle, for future examinations. The medial head of the gastrocnemius at that level was imaged for all future examinations. Once an appropriate image was obtained, of the contrast was injected. Recording commenced immediately upon injection and was concluded when no further contrast was visible. Ultrasonography was performed using a Linear L9-3 Transducer with an iU22 System (Phillips Inc., Andover, MA).

### Graded treadmill exercise

After baseline testing, subjects were asked to perform standardized treadmill walking. This began at the maximal speed and grade that had been determined during initial evaluation to result in the onset of claudication. Patients were asked to walk for 10 min or until pain limited further ambulation, whichever occurred first.

To achieve a comparable relative level of exertion, control subjects walked at a grade and speed combination that allowed achievement and maintenance of 60% heart rate reserve for a 10 min walking protocol.

### Image analysis

After image acquisition, data were transferred to an off-line computer and analysed using QLab (Philips Ultrasound, Andover, MA). A region of interest was selected with superficial bound of ~0.5 cm deep to the gastrocnemius fascia, as visualized on ultrasound; deep bound of 3.5 cm below fascia; and lateral bounds of the visualized portion of the muscle. Acoustic intensity (AI) of every frame within this region of interest was determined using QLab. AI is based upon decibel intensity of signal return and is a validated surrogate for perfusion in contrast ultrasound studies [5,6,23,24].

**Figure 1:**
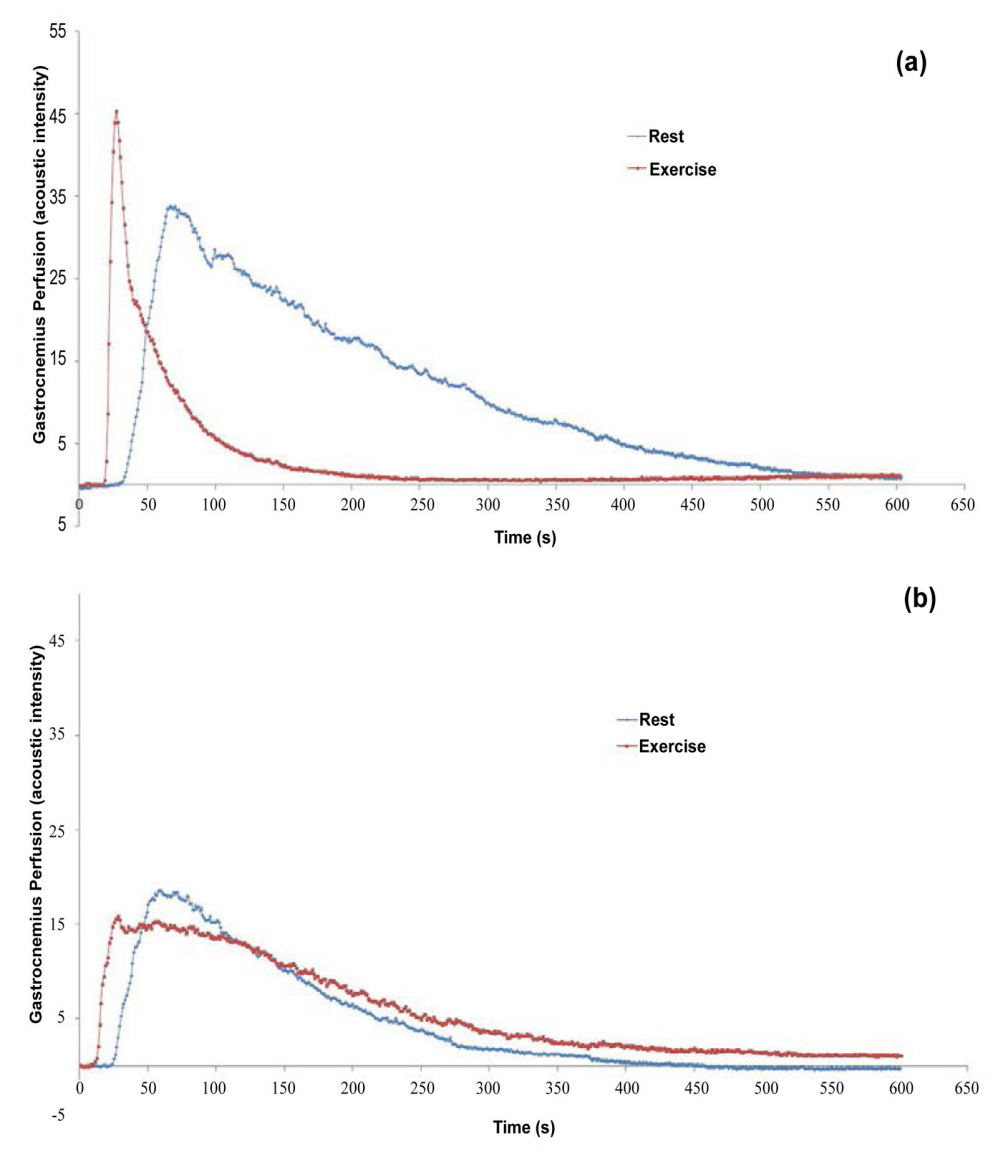
Representative intensity versus time plots of gastrocnemius perfusion in normal subjects (a) and PAD patients (b) at rest (blue line) and after exercise (red line).

**Figure 2:**
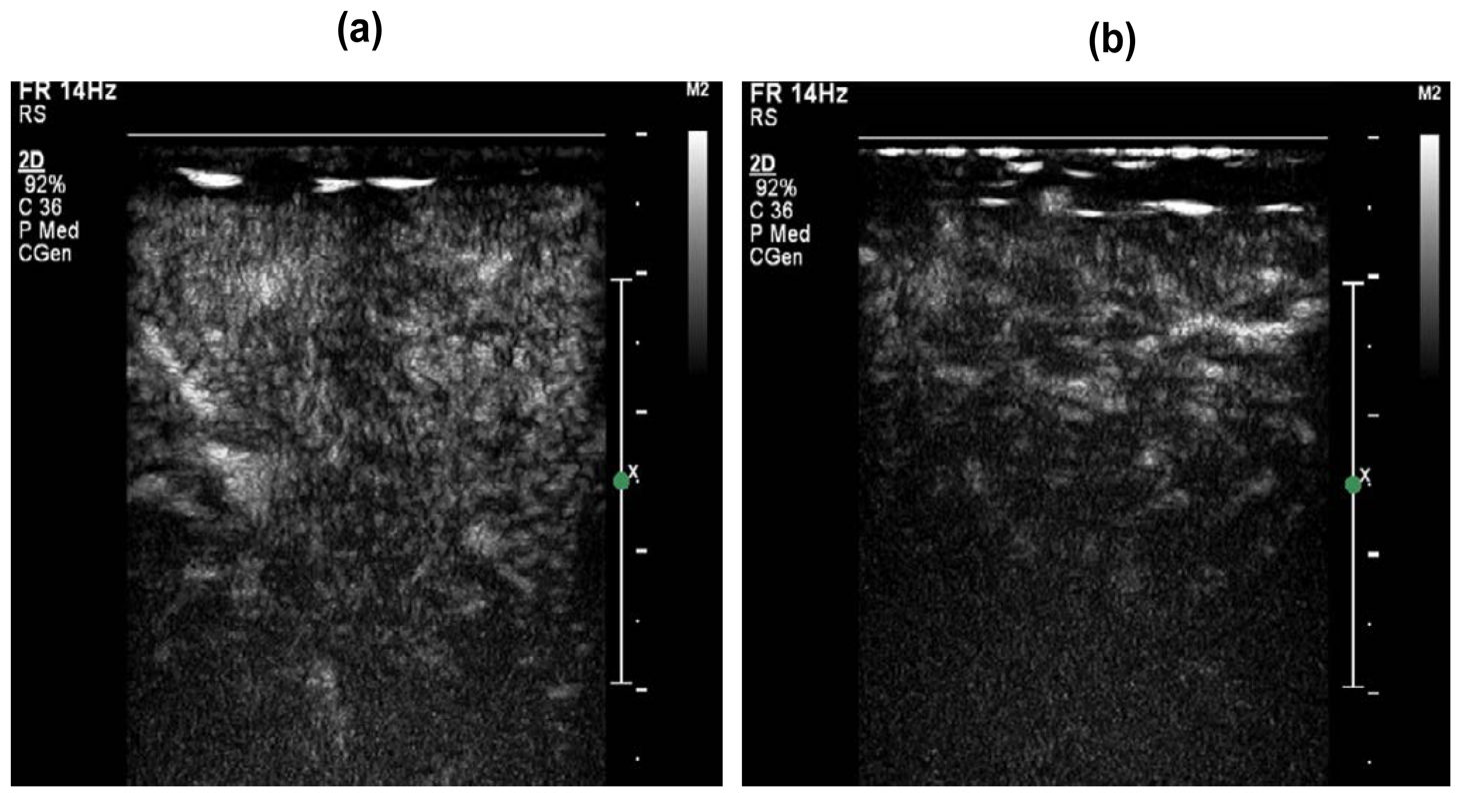
Representative contrast-enhanced ultrasound images at the time of exercise-induced peak gastrocnemius perfusion in normal subjects (a) and PAD patients (b).

A graph of AI versus Time was produced from the QLab analysis; examples and representative CEUS images are shown in Figures 1 and 2. Two variables were derived from these graphs and images. First, the degree of peak perfusion (PEAK), the maximal AI recorded within the ROI, and second, the time to peak perfusion (TTP), or the length of interval between the first appearance of contrast and the frame of greatest AI. These variables have been used in past studies of perfusion in both bolus and constant-infusion protocols [12–15,21,22,24–26].

## Statistical Analysis

Four dependent variables were recorded for each patient: TTP and PEAK at rest (TTP_B_ and PEAK_B_) and after exercise (TTP_X_ and PEAK_X_). The mean and standard deviations of each of these four variables were calculated for patients with PAD (PAD) and normal control (Normal).

Statistical analysis was performed using SPSS Version 24.0 (IBM Corp, Armonk, NY). Welch’s t-test for homoscedastic, unequal sample sizes was calculated. Significance was set at *p* ≤ 0.05.

Comparison was made within NORMAL and PAD groups between resting and exercise measures and between NORMAL and PAD groups between resting and exercise measures. Finally, the magnitude of change (TTX_(B-X)_ and PEAK_(B-X)_) was compared between NORMAL and PAD groups.

## Results

Major findings are listed in Table 1.

Thirteen patients with intermittent claudication were recruited. All were males with an average age of 66 ± 6.4 years and an average ABI of 0.60 ± 0.10 in the index, limiting lower extremity. Eight volunteer control subjects were enrolled, all of whom were males with an average age of 43 ± 12.7 years and palpable dorsalis pedis and posterior tibial pulses bilaterally.

**Figure 3:**
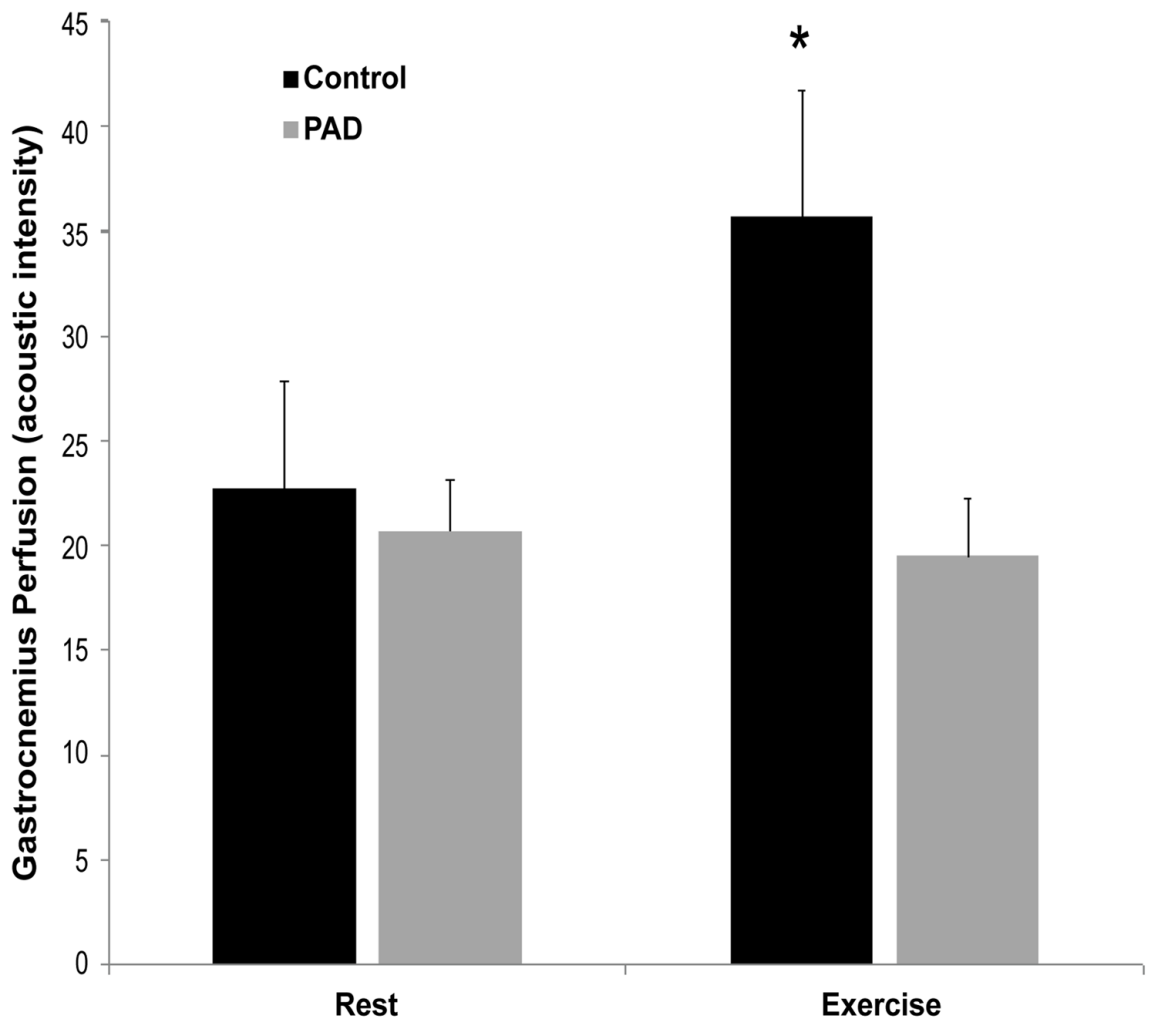
Intensity of peak perfusion before and after exercise. At rest, no significant difference in perfusion is seen between Control and PAD subjects. After exercise, Control subjects demonstrate significantly greater perfusion. Data are means ± SEM. *Significant difference, p<0.05.

**Figure 4:**
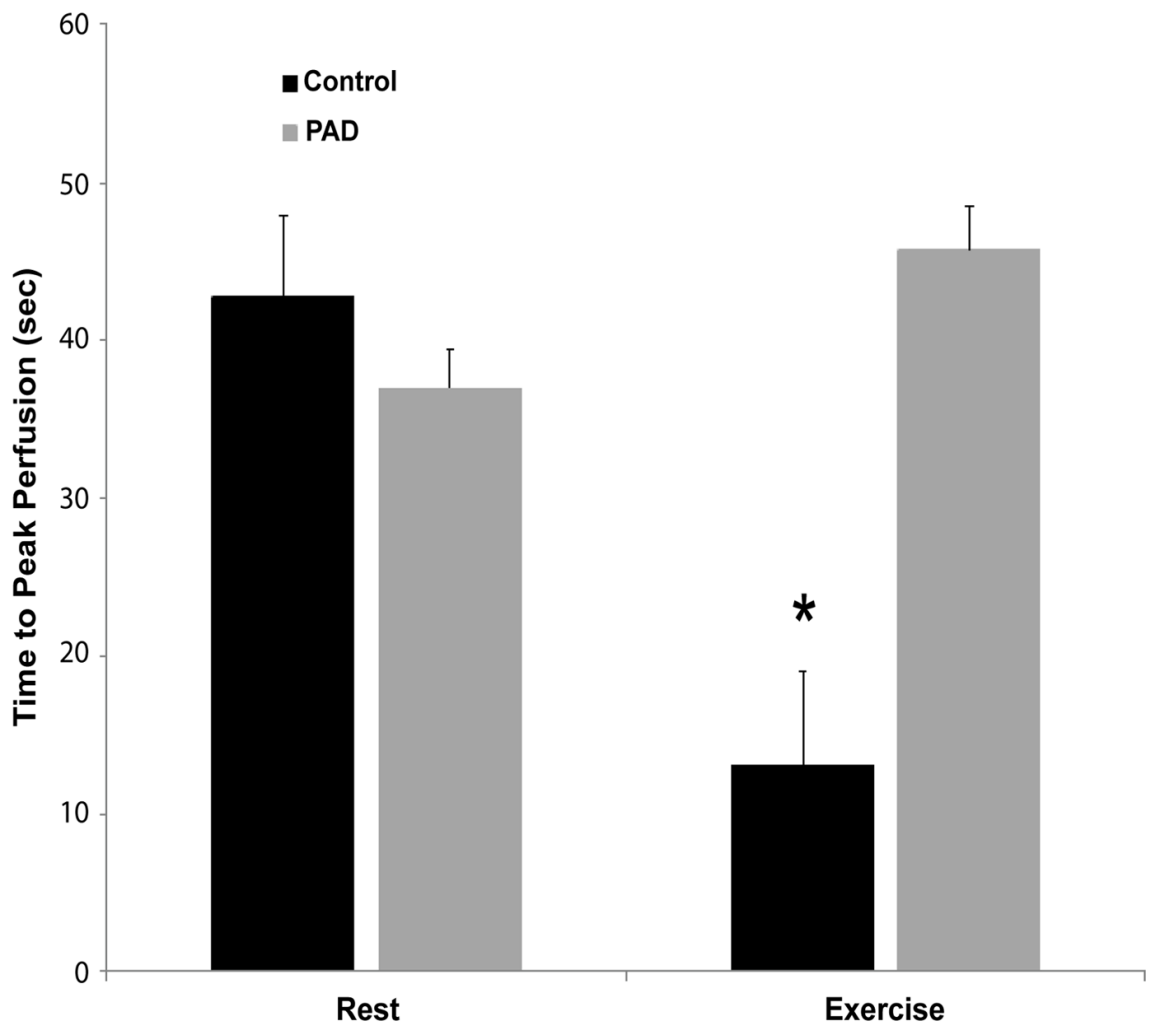
Time to peak perfusion before and after exercise. At rest, no significant difference in perfusion is seen between Control and PAD subjects. After exercise, Control subjects demonstrate significantly faster achievement of peak perfusion. Data are means ± SEM. *Significant difference, p<0.05.

Comparing normal subjects with PAD patients at rest, no significant differences were found in either TTP_B_ (*p*=0.39) or PEAK_B_ (*p*=0.71). After exercise, however, there was significantly lower TTP_X_ (*p*=0.04) and significantly higher PEAK_X_ (*p*=0.02) in normal subjects compared to PAD patients (Figures 3 and 4). In subjects without PAD, treadmill exercise was associated with a significant decrease in TTP (*p*<0.01) and a significant increase in PEAK (*p*=0.02). In comparison, PAD patients did not demonstrate any significant alteration in either TTP (p=0.49) or PEAK (p=0.67) in response to exercise testing.

**Figure 5:**
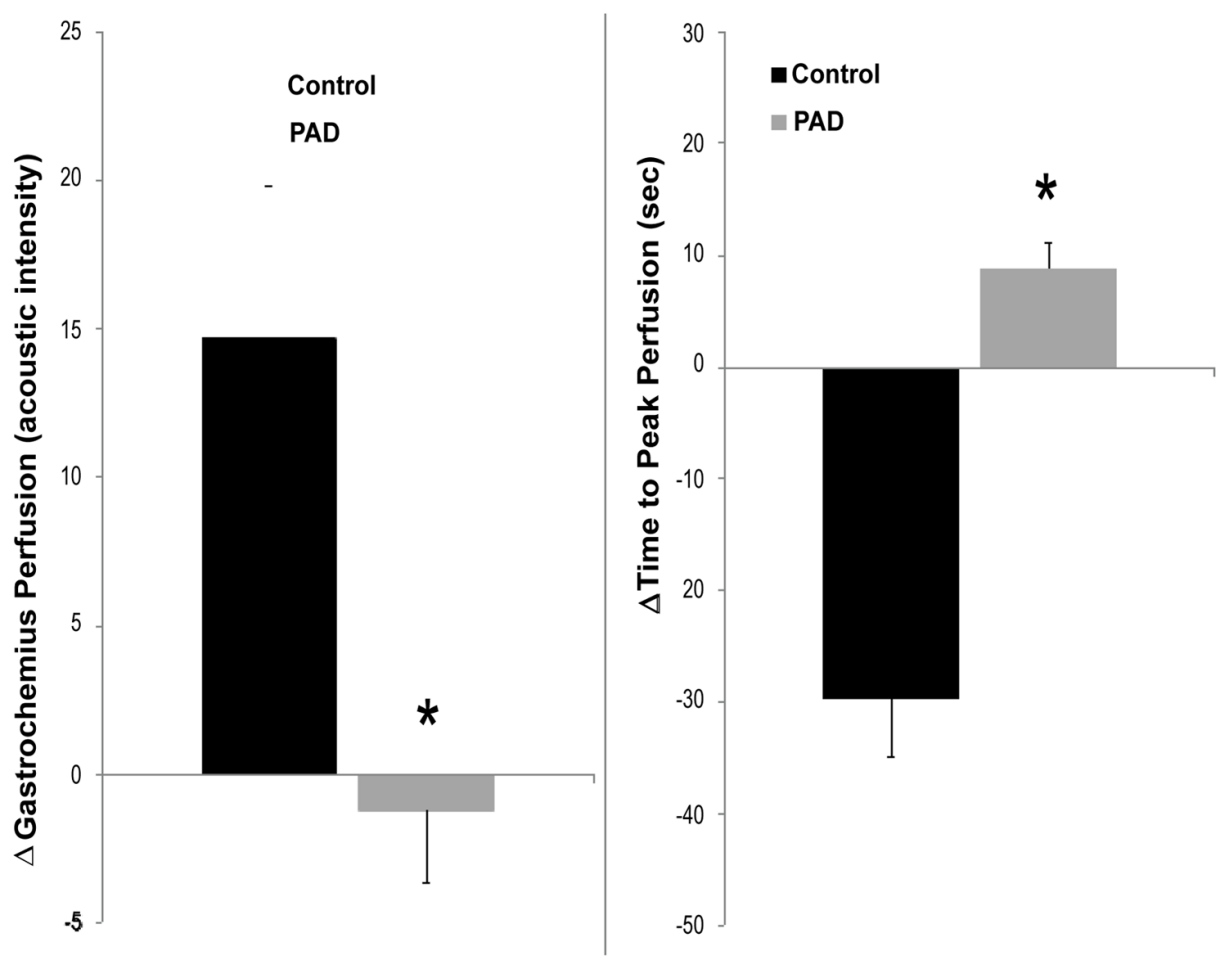
Exercise-induced changes in intensity of perfusion (left) and time to peak perfusion (right) in Control and PAD subjects. Exercise had significantly greater effects on both measures in control subjects. “Data are means +/− SEM. *Significant difference, p:0.05.”

Finally, comparing the exercise-induced alterations in each group, significant differences were seen between control patients and PAD patients, with greater changes in both time to peak and intensity of peak perfusion seen in normal subjects than in PAD patients (Figure 5).

## Discussion

The noninvasive assessment of perfusion deficits in PAD patients with mild claudication presents a significant clinical challenge. The most commonly used noninvasive study, the single-level ankle-to-brachial index, is frequently normal in mild claudicants [18,19]. While exercise ABI is more sensitive, both resting and exercise studies are inherently flawed as they measure the intravascular pressure of named arteries as a surrogate for lower extremity tissue perfusion, which depends on more distal arterial branches and arterioles [27]. The ankle to brachial index can be normal and pedal pulses palpable while proximal resting ischemia and even gangrene is present [28–31]. More frequently, the presence of medial calcific disease, common in people with diabetes, results in vessel rigidity that falsely elevates the ABI, rendering this measure extremely unreliable [32].

There is currently no widely-used, minimally invasive method of measuring actual end-organ perfusion of lower extremity musculature. Investigations into the use of near-infrared spectroscopy are confounded by effort-independent skin and subcutaneous perfusion [33–35]. The clinical relevance of perfusion deficits of the gastrocnemius is significant, as even mild claudication results in neural and muscular atrophy and loss of muscle function [36].

Contrast-enhanced ultrasound assessment of perfusion has been validated experimentally in multiple contexts, including lower extremity skeletal muscle, but to date, CEUS investigations in PAD have been primarily in subjects at rest [12,26]. The difficulty in assessing perfusion deficits in claudicants, in whom ischemia is only present with effort, has spurred some investigators to use post-occlusive reactive hyperemia to simulate the changes induced by exercise. Such studies have been performed in healthy volunteers as well as PAD patients with and without diabetes [15,20,21]. The results of these have been validated against exercise testing with ABI measurement [22]. An additional study substituted calf-raises for walking [12]. Either method, however, correlates perfusion with subjective claudication pain or objective measures of function, an important measure considering that claudication pain and function are the primary indication for surgical treatment. The clinical utility of an outcome measure that does not take into account the patient’s clinical result is uncertain.

We present findings linking both subjective and objective measures of claudication and mobility function with assessment of lower extremity perfusion using a simple and easily replicated protocol within the capabilities of any existing clinical vascular laboratory. We demonstrate that claudicants are unable to significantly increase the intensity of gastrocnemius perfusion or the time to maximal perfusion after exercise. This supports the classical demand-ischemia model of intermittent claudication in which maximal gastrocnemius vasodilation maintains sufficient perfusion at rest; no reserve remains, however, to accommodate further demand. The treadmill exercise therefore results in ischemia. An inability to compensate for increased demand during exercise results in unchanged perfusion and symptomatic ischemia [37]. Normal subjects, in comparison, show significant increases in perfusion intensity and decreases in time to peak. This is also consistent with understood exercise physiology [38–40].

Of note, there were no observed differences in resting perfusion between normal subjects and PAD patients. This appears to conflict with the abnormally low ABI in the PAD patients compared to the palpable pedal pulses, a reliable indicator of normal ABI, in the normal subjects [41]. One possible explanation is that the greater metabolic efficiency of normal subjects results in a reduced resting demand by normal tissue [42,43]. It is possible that in the setting of decreased perfusion, atrophy and loss of muscle volume continue until a baseline density of perfusion is met in other words, normalization of muscle mass for decreased perfusion.

Despite this similarity in resting perfusion, walking exercise causes significant enhancement in perfusion in normal subjects but not PAD patients, resulting in significantly decreased time to peak perfusion and increased intensity of perfusion in normal subjects. While our findings are consistent with cardiovascular exercise physiology, several opportunities exist for improvement of our methodology in future studies. Our normal subjects were not age or comorbidity matched, but was volunteers. This was intentional, as we sought, for maximum clinical relevance, to contrast perfusion in PAD patients with models of optimal physiology. Future work, must take into account age related changes and cardiac comorbidity, as both systolic and diastolic function affect the passage of contrast from a peripheral vein to the extremity arterial tree [44]. Moreover, as mentioned above, the incidence of muscle atrophy among PAD patients is substantial, and correlation of relative muscle loss to perfusion deficit may reveal significant physiologic information [36,43,47]. Comparison of CEUS findings with biopsy-derived capillary density and muscle ultrastructure may hold these answers.

Our findings support the utility of a simplified protocol for assessment of exercise-induced changes in perfusion and correlation with subjective and objective measures of claudication. Further investigation into the clinical utility of this method is warranted.

## Figures and Tables

**Table 1:** Time to peak perfusion (TTP) and intensity of peak perfusion (PEAK) at rest (B) and after exercise (X) in control subjects (NORMAL) and PAD patients (PAD). Significant differences are found between NORMAL and PAD in both time to peak and intensity of peak perfusion after exercise, but not at rest. Significant changes in time to peak and intensity of peak perfusion in NORMAL, but not in PAD.

	NORMAL	PAD	*p*
**TTPB**	42.7	37	*p*=0.39
**PEAKB**	22.7	20.7	*p*=0.71
**TTPX**	13	45.7	*p*=0.04
**PEAKX**	35.6	19.4	*p*=0.02
**TTP(B-X)**	*p*<0.01	*p*=0.49	-
**PEAK(B-X)**	*p*=0.02	*p*=0.67	-
